# State of energy estimation of lithium-ion battery based on long short-term memory optimization Adaptive Cubature Kalman filter

**DOI:** 10.1371/journal.pone.0306165

**Published:** 2024-07-10

**Authors:** Enguang Hou, Heyan Song, Zhen Wang, Jingshu Zhu, Jiarui Tang, Gang Shen, Jiangang Wang

**Affiliations:** 1 School of Rail Transportation, Shandong Jiao Tong University, Jinan, China; 2 School of Electrical Engineering, Shandong University, Jinan, China; 3 Jinan Park Development Service Center, Jinan, China; Buckinghamshire New University - High Wycombe Campus: Buckinghamshire New University, UNITED KINGDOM

## Abstract

State of energy (SOE) is an important parameter to ensure the safety and reliability of lithium-ion battery (LIB) system. The safety of LIBs, the development of artificial intelligence, and the increase in computing power have provided possibilities for big data computing. This article studies SOE estimation problem of LIBs, aiming to improve the accuracy and adaptability of the estimation. Firstly, in the SOE estimation process, adaptive correction is performed by iteratively updating the observation noise equation and process noise equation of the Adaptive Cubature Kalman Filter (ACKF) to enhance the adaptive capability. Meanwhile, the adoption of high-order equivalent models further improves the accuracy and adaptive ability of SOE estimation. Secondly, Long Short-term Memory (LSTM) is introduced to optimize Ohmic internal resistance (OIR) and actual energy (AE), further improving the accuracy of SOE estimation. Once again, in the process of OIR and AE estimation, the iterative updating of the observation noise equation and process noise equation of ACKF were also adopted to perform adaptive correction and enhance the adaptive ability. Finally, this article establishes a SOE estimation method based on LSTM optimized ACKF. Validate the LSTM optimized ACKF method through simulation experiments and compare it with individual ACKF methods. The results show that the ACKF estimation method based on LSTM optimization has an SOE estimation error of less than 0.90% for LIB, regardless of the SOE at 100%, 65%, and 30%, which is more accurate than the SOE estimation error of ACKF alone. It can be seen that this study has improved the accuracy and adaptability of LIB’s SOE estimation, providing more accurate data support for ensuring the safety and reliability of lithium batteries.

## 1. Introduction

With the environmental pollution caused by the combustion of fossil fuels, the development of green and sustainable energy storage devices is of great significance for the utilization of renewable energy. As a new type of energy storage equipment that provides electricity for the world, lithium batteries have received more and more attention [1–4].

State of energy (SOE) is an important parameter of battery management system (BMS), which is the ratio of remaining available energy to the maximum available energy [5, 6], and is one of the most critical parameters in BMS. In order to improve the performance of electric vehicle BMS, a high-precision SOE estimation algorithm is needed [7].

**Fig 1:**
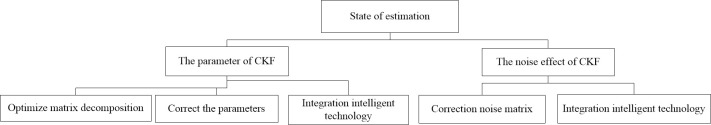
SOE estimation method.

However, accurate SOE estimation is challenging because of erratic battery dynamics and SOE variation with current, temperature, operating conditions, etc. In recent years, Cubature Kalman Filter (CKF) has been widely used in SOE estimation of LIBs due to its good dynamic tracking ability. Arasaratnam and Haykin [8, 9] first proposed a CKF method of volume Kalman filtering based on Cubature integral transformation. The research of CKF in LIBs state estimation is mainly divided into two directions(as shown in Fig 1): one is to optimize the parameters of CKF; The second is to optimize the noise effect of CKF.

In terms of parameter optimization, the first step is to optimize matrix decomposition. Li et al. [10] proposed an improved CKF algorithm, which implements the diagonalization decomposition of the covariance matrix and a strong tracking filter. In Ref. [11], three typical matrix decomposition strategies, namely, singular value decomposition (SVD), UR decomposition, and LU decomposition are introduced, to replace the Cholesky decomposition in the traditional CKF.

In terms of parameter optimization, the second research direction is to correct the parameters of LIBs. In Ref. [12], forgetting factor recursive least squares (FFRLS), optimal bounding ellipsoid (OBE), and linear Kalman filter (LKF) are discussed, and the OBE algorithm is more suitable. In Ref. [13], an efficient method of parameter identification for LIBs using CKF and least square with gradient correction is proposed. Li et al. [14] introduced the constraint condition of the pneumatic principle to replace the temperature correction coefficient, which can realize the fast convergence of SOC. Li et al. [15] the vector forgetting factor recursive least squares method is utilized for model parameter online identification.

Finally, intelligent technology is integrated into the parameter optimization process. Wang et al. [16] combined the H ∞ filter with SVD-CKF to solve the problem of decreased SOC estimation accuracy caused by temperature changes. Ma et al. [17] used the generalized maximum correlation criterion and fixed-point iteration method to enhance the robustness of the filter and better adapt to various complex situation. Fu et al. [18] introduced a new type of weighted multi-innovation Cubature Kalman filter (WMICKF). This filter can innovate vector weighting calculations based on error distribution and time distribution, thereby achieving SOC estimation. Compared to traditional methods, WMICKF has higher estimation accuracy and better robustness, and can effectively handle SOC estimation problems in complex and ever-changing environments. Song et al. [19] proposed a new joint support vector machine- cubature Kalman filter method.

In order to study the noise effect of CKF optimization, the noise matrix is modified first. Liu et al. [20] found that the Sage-Husa estimator can timely grasp the statistical characteristics of process and measurement noise, and make corrections to them. Experiments have shown that this algorithm has strong robustness to the initial error of SOC. Wang et al. [21] proposed a novel state noise matrix self-tuning CKF algorithm based on the optimal model. The experimental results show that this improved CKF algorithm performs well in tracking the minimum SOC envelope of parallel battery module. During the voltage plateau period, the estimated SOC error remains stable within 1.2%, and at the end of discharge, it remains stable within 4.3%. These data fully demonstrate the effectiveness of the proposed method. Wang et al. [22] proposed a novel variable forgetting factor recursive least square (VFFRLS) noise adaptive CKF algorithm based on the VFFRLS algorithm to cope with changes in model parameters. In Ref. [23], Adaptive noise recognition combined with dual Kalman filters to achieve higher robustness and computational efficiency.

In the same way as parameter optimization, intelligent technology is also incorporated in noise optimization. In Ref. [24], the second-order resistor capacitor equivalent circuit model and the VFFRLS online parameter identification method were adopted, and a fuzzy adaptive controller was proposed based on this. The purpose of this controller is to improve the convergence speed of SOC estimation for steady-state Kalman filter. Tian et al. [25] elaborates on a method of integrating long- short term memory LSTM networks with ACKF to estimate battery state more accurately and stably.

In summary, the integration of intelligent technology and CKF has become a development trend in the future. The integration of intelligent technology and CKF will further improve the accuracy and adaptive characteristics of SOE estimation.

Validate the LSTM optimized ACKF method through simulation experiments and compare it with individual ACKF methods. The results indicate that the proposed method can significantly improve the estimation accuracy of SOE.

State of energy estimation of LIBs based on long short-term memory optimization ACKF has been proposes in the paper, and the superiority of method is verified. There are three original contributions as follows:

1. In the process of SOE estimation, the observation noise equation and process noise equation of ACKF are updated iteratively to make adaptive correction and enhance the adaptive ability.
2. The LSTM is introduced to optimize the Ohmic internal resistance (OIR) and actual energy (AE), and further improve the accuracy of SOE estimation.
3. In the process of OIR and AE estimation, the observation noise equation and process noise equation of ACKF are updated iteratively to make adaptive correction and enhance the adaptive ability.

## 2. Estimation model of SOE

### 2.1. SOE

The SOE of LIB: the ratio of the remaining energy to the nominal energy, namely:

$$S_{ek+1}=S_{ek}-\frac{U_{k}\Delta t}{E}i_{k}$$ (01)

where, *S*_*ek*_, *i*_*k*_, and *U*_*k*_ are the SOE, current, and working voltage of a LIB at *k* time in discrete state; *E* is nominal energy of a LIB; Δ*t* is the sampling period.

### 2.2. SOE estimation model based on TRCEM

In order to simulate the charge and discharge characteristics of lithium-ion batteries more accurately, in the process of selecting the equivalent circuit model, not only the polarization of lithium-ion batteries, but also the complexity and practicability of the equivalent model should be considered. Based on the consideration of accuracy, complexity, and practicability, third-order resistor-capacitance equivalent model (TRCEM) is chosen in this paper.

**Fig 2:**
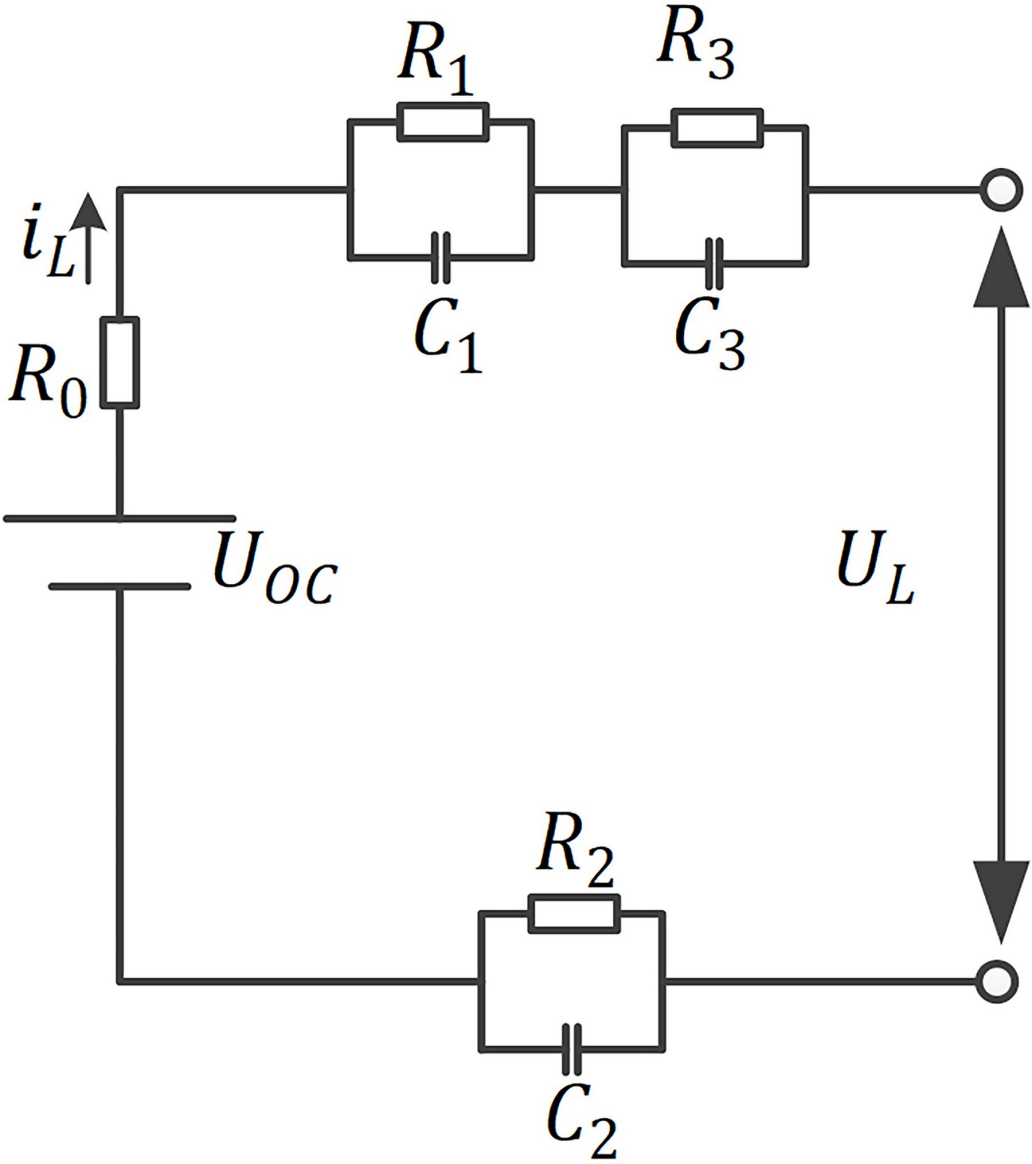
The TRCEM of SOE estimation.

In Fig 2, *U*_*oc*_, *U*_*L*_, *i*_*L*_, and *R*_0_ are the open circuit voltage (OCV), working voltage, charge/discharge current, and Ohmic internal resistance of LIB; *R*_1_, *R*_2_, and *R*_3_ is the Ohmic polarization resistance, electrochemical polarization internal resistance, and the internal resistance of concentration difference polarization; *C*_1_, *C*_2_, and *C*_3_ is the Ohmic polarization capacitance, electrochemical polarization capacitance, and concentration differential polarization capacitance; *U*_1_, *U*_2_, and *U*_3_ are the voltages at both ends of capacitor *C*_1_, *C*_2_, and *C*_3_ respectively; *τ*_1_ = *R*_1_*C*_1_, *τ*_2_ = *R*_2_*C*_2_, *τ*_3_ = *R*_3_*C*_3_, time constant.

According to Fig 2, the discrete state equation of LIB’s TRCEM is as follows:

$$[\begin{array}{l} S_{ek+1}\\ U_{k+1}^{R_{1}C_{1}}\\ U_{k+1}^{R_{2}C_{2}}\\ U_{k+1}^{R_{3}C_{3}} \end{array}]=[\begin{array}{cccc} 1 & 0 & 0 & 0\\ 0 & \exp(-\frac{\Delta t}{\tau_{1}}) & 0 & 0\\ 0 & 0 & \exp(-\frac{\Delta t}{\tau_{2}}) & 0\\ 0 & 0 & 0 & \exp(-\frac{\Delta t}{\tau_{3}}) \end{array}]\cdot [\begin{array}{c} S_{ek}\\ U_{k}^{R_{1}C_{1}}\\ U_{k}^{R_{2}C_{2}}\\ U_{k}^{R_{3}C_{3}} \end{array}]+[\begin{array}{c} -\frac{U_{k}\Delta t}{E}\\ R_{1}(1-\exp(-\frac{\Delta t}{\tau_{1}}))\\ R_{2}(1-\exp(-\frac{\Delta t}{\tau_{2}}))\\ R_{3}(1-\exp(-\frac{\Delta t}{\tau_{3}})) \end{array}]\cdot i_{k}+q_{k}$$ (02)

According to Fig 2, the discrete observation equation of LIB’s TRCEM is as follows:

$$U_{k}=[\frac{d(U_{oc}(S_{e}))}{dS_{e}}{|}_{S_{e}=S_{ek}}-1-1-1]∙\left[\begin{array}{c} S_{ek}\\ U_{k}^{R_{1}C_{1}}\\ U_{k}^{R_{2}C_{2}}\\ U_{k}^{R_{3}C_{3}} \end{array}\right]-i_{k}R_{0}$$ (03)

As

$$A_{k}=[\begin{array}{cccc} 1 & 0 & 0 & 0\\ 0 & \exp(-\frac{\Delta t}{\tau_{1}}) & 0 & 0\\ 0 & 0 & \exp(-\frac{\Delta t}{\tau_{2}}) & 0\\ 0 & 0 & 0 & \exp(-\frac{\Delta t}{\tau_{3}}) \end{array}],\;B_{k}=[\begin{array}{c} -\frac{U_{k}\Delta t}{E}\\ R_{1}(1-\exp(-\frac{\Delta t}{\tau_{1}}))\\ R_{2}(1-\exp(-\frac{\Delta t}{\tau_{2}}))\\ R_{3}(1-\exp(-\frac{\Delta t}{\tau_{3}})) \end{array}],\;x_{k}=[\begin{array}{c} S_{ek}\\ U_{k}^{R_{1}C_{1}}\\ U_{k}^{R_{2}C_{2}}\\ U_{k}^{R_{3}C_{3}} \end{array}],$$

$${u_{k}=i}_{k},C_{k}=[\frac{d(U_{oc}(S_{e}))}{dS_{e}}{|}_{S_{e}=S_{ek}}-1-1-1].$$

So

$$f(x_{k},u_{k})=A_{k}x_{k}+B_{k}u_{k}$$ (04)

$$g(x_{k},u_{k})=C_{k}x_{k}-R_{0,k}u_{k}$$ (05)

where, $U_{k}^{R_{1}C_{1}},\;U_{k}^{R_{2}C_{2}},\;U_{k}^{R_{3}C_{3}}$, and $U_{k+1}^{R_{1}C_{1}},\;U_{k+1}^{R_{2}C_{2}},\;U_{k+1}^{R_{3}C_{3}}$ are the estimated voltage values of *R*_1_, *R*_2_, *R*_3_ at *k* and *k+1* time in discrete state; *q*_*k*_, *γ*_*k*_ are independent system noises; *U*_*oc*_(*S*_*e*_) is the OCV of a LIB corresponding to the SOE value of a LIB at the *k* time in discrete state.

### 2.3. Model parameter identification

The *U*_*L*_, *i*_*L*_, and *U*_OC_ of the LIB collected through the charge/discharge test using test equipment (BTS20). Model parameter identification based on the least square method is not repeated in this article because the method is described in detail in Refs. [26]. Both *U*_*OC*_ and *R*_0_ adopt the off-line identification method [6, 26]. The initial values of parameters identified in this paper are shown in Table 1.

**Table 1:** The initial values of parameters of identification.

*R*_0_/Ω	*R*_1_/Ω	*R*_2_/Ω	*R*_3_/Ω	*C*_1_/F	*C*_2_/F	*C*_3_/F	t/s	E/wh
0.0011	0.0022	0.0058	0.0036	541.87	1029.76	795.50	3400	192

## 3. SOE estimation

In this article, ACKF is used to carry out SOE estimation research based on the TRCEM, and the estimation accuracy and adaptability characteristics are compared and analyzed [27].

### 3.1. SOE estimation based on ACKF

From Formulas (04) and (05), the state and observation formulas:

$$x_{k+1}=f(x_{k},\theta_{k})+q_{k}$$ (06)

$$y_{k+1}=g(x_{k},u_{k},\theta_{k})+\gamma_{k}$$ (07)

where, *θ*_*k*_ = [*R*_0,*k*_, *E*_*k*_]^*T*^, *R*_0,*k*_ and *E*_*k*_ are the state variable OIR and AE; *u*_*k*_ and *y*_*k*_ are the input and observation variables of the system, which are the current and the working voltage of a LIB. *q*_*k*_ *and γ*_*k*_ are the zero-mean Gaussian white noise; the error covariance matrices of *q*_*k*_ *and γ*_*k*_ are *Q*_*k*_ *and R*_*k*_.

The ACKF algorithm flow:

Step 1: Initialize *x*_*k*_:

$${\hat{x}}_{0}=E(x_{0})$$ (08)

$${\hat{P}}_{0}=E[(x_{0}-{\hat{x}}_{0}){\left(x_{0}-{\hat{x}}_{0}\right)}^{T}$$ (09)

Step 2: Time update of *x*_*k*_:

Cubature Points,

$$S_{k-1}=\sqrt{{\hat{P}}_{k-1}}$$ (10)

$$\chi_{k-1}^{i}={\hat{x}}_{k-1}+S_{k-1}\xi_{i}$$ (11)

$$\chi_{k}^{i}=f(\chi_{k-1}^{i})+q_{k-1},i=1,2,\ldots ,m$$ (12)

State prediction,

$$x_{k}=\frac{1}{m}\sum_{i=1}^{m}\chi_{k}^{i}$$ (13)

Where $\xi_{i}=\sqrt{\frac{m}{2}}{\left[1\right]}_{i},i=1,2,\ldots ,m=2n,\;\omega_{i}=\frac{1}{m},i=1,2,\ldots ,m=2n$.

[1] indicates that *n* is the set of points in *u* space, i.e.:

$$\left[(\begin{array}{c} 1\\ 0\\ \vdots \\ 0 \end{array})\ldots (\begin{array}{c} 0\\ 0\\ \vdots \\ 1 \end{array})(\begin{array}{c} -1\\ 0\\ \vdots \\ 0 \end{array})\ldots (\begin{array}{c} 0\\ 0\\ \vdots \\ -1 \end{array})\right]$$

State prediction covariance:

$$P_{k}=\frac{1}{m}\sum_{i=1}^{m}\chi_{k}^{i}{\left(\chi_{k}^{i}\right)}^{T}-x_{k}{\left(x_{k}\right)}^{T}+Q_{k-1}$$ (14)

Step 3: Measurement update of *y*_*k*_:

Cubature Points,

$$S_{k}=\sqrt{P_{k}}$$ (15)

$${\hat{\chi }}_{k}^{i}=x_{k}+S_{k}\xi_{i}$$ (16)

$$y_{k}^{i}=h({\hat{\chi }}_{k}^{i})+\gamma_{k},i=1,2,\ldots ,m$$ (17)

Observation prediction:

$${\hat{y}}_{k}=\frac{1}{m}\sum_{i=1}^{m}y_{k}^{i}$$ (18)

The Kalman gain is as follows:

$$P_{y,k}=\frac{1}{m}\sum_{i=1}^{m}(y_{k}^{i}-{\hat{y}}_{k}){\left(y_{k}^{i}-{\hat{y}}_{k}\right)}^{T}+R_{k}$$ (19)

$$P_{xy,k}=\frac{1}{m}\sum_{i=1}^{m}({\hat{\chi }}_{k}^{i}-x_{k}){\left(y_{k}^{i}-{\hat{y}}_{k}\right)}^{T}$$ (20)

$$K_{k}=P_{xy,k}P_{y,k}^{-1}$$ (21)

The optimal estimation of state variables is as follows:

$${\hat{x}}_{k}=x_{k}+K_{k}[y_{k}-{\hat{y}}_{k}]$$ (22)

The optimal estimate of the covariance is as follows:

$${\hat{P}}_{k}=P_{k}-K_{k}P_{y,k}K_{k}^{T}$$ (23)

State error and observation error:

$$\varepsilon_{x,k}={\hat{x}}_{k}-x_{k}$$ (24)

$$\varepsilon_{y,k}={\hat{y}}_{k}-y_{k}$$ (25)

Step 4: Process noise covariance equation is as follows:

$$q_{k}=(1-d_{k})q_{k-1}+d_{k}\varepsilon_{x,k}$$ (26)

$$Q_{k}=(1-d_{k})Q_{k-1}+d_{k}[K_{k}\varepsilon_{y,k}\varepsilon_{y,k}^{T}K_{k}^{T}+P_{k}-A_{k-1}{\hat{P}}_{k-1}A_{k-1}^{T}]$$ (27)

Step 5: Observation noise covariance equation is as follows:

$$\gamma_{k}=(1-d_{k})\gamma_{k-1}+d_{k}\varepsilon_{y,k}$$ (28)

$$R_{k}=(1-d_{k})R_{k-1}+d_{k}\varepsilon_{y,k}\varepsilon_{y,k}^{T}-{C_{k}P_{y,k}C}_{k}^{T}]$$ (29)

where $d_{k}=\frac{1-b}{1-b_{k}}$, *k* = 1,2,⋯,*n*, *b* is the forgetting factor,0<*b*<1; *x*_*k*_, *y*_*k*_, and *P*_*k*_ are the estimation of the state, estimation of observation value variable, and estimation of the error covariance; ${\hat{x}}_{k},\;{\hat{y}}_{k}$, and ${\hat{P}}_{k}$ are optimal estimation of the state variable, actual observation value variable, and optimal estimation of the error covariance.

According to Eqs (2), (3), (13),

$${\hat{x}}_{k}=\left[\begin{array}{c} {\hat{S}}_{ek}\\ {\hat{U}}_{k}^{R_{1}C_{1}}\\ {\hat{U}}_{k}^{R_{2}C_{2}}\\ {\hat{U}}_{k}^{R_{3}C_{3}} \end{array}\right]$$ (30)

where ${\hat{S}}_{ek}$ is the optimal value of SOE based on ACKF.

### 3.2. OIR and AE estimation based on ACKF

The state and observation formulas of the system with the newly added state parameters:

$$\theta_{k+1}=\theta_{k}+q_{\theta ,k}$$ (31)

$$z_{k+1}=g(x_{k},u_{k},\theta_{k})+\gamma_{\theta ,k}$$ (32)

where *q*_*θ*,*k*_ is the noise on the input variable, and it is the zero-mean Gaussian white noise; *γ*_*θ*,*k*_ is the noise on the output variable, and it is the zero-mean Gaussian white noise; the error covariance matrices of *q*_*θ*,*k*_ *and γ*_*θ*,*k*_ are *Q*_*θ*,*k*_ *and R*_*θ*,*k*_; the state variable *θ* is estimated based on ACKF algorithm, and the estimated values of the LIB’s OIR and actual energy (AE) are calculated. In order to improve the accuracy, the error between the actual value and the estimated value of the working voltage is optimized.

The ACKF algorithm flow:

Step 1: Initialize *θ*_*k*_:

$${\hat{\theta }}_{0}=E(\theta_{0})$$ (33)

$$P_{\theta ,0}=E[(x_{0}-{\hat{x}}_{0}){\left(x_{0}-{\hat{x}}_{0}\right)}^{T}]$$ (34)

Step 2: Time update of *θ*_*k*_:

Cubature Points,

$$S_{\theta ,k-1}=\sqrt{{\hat{P}}_{\theta ,k-1}}$$ (35)

$$\chi_{\theta ,k-1}^{i}={\hat{\theta }}_{k-1}+S_{\theta ,k-1}\xi_{\theta ,i}$$ (36)

$$\chi_{\theta ,k}^{i}=f(\chi_{\theta ,k-1}^{i})+q_{\theta ,k-1},i=1,2,\ldots ,m$$ (37)

State prediction,

$$\theta_{k}=\frac{1}{m}\sum_{i=1}^{m}\chi_{\theta ,k}^{i}$$ (38)

Where $\xi_{\theta ,i}=\sqrt{\frac{m}{2}}{\left[1\right]}_{i},i=1,2,\ldots ,m=2n,\;\omega_{\theta ,i}=\frac{1}{m},i=1,2,\ldots ,m=2n$.

State prediction covariance:

$$P_{\theta ,k}=\frac{1}{m}\sum_{i=1}^{m}\chi_{\theta ,k}^{i}{\left(\chi_{\theta ,k}^{i}\right)}^{T}-\theta_{k}{\left(\theta_{k}\right)}^{T}+Q_{\theta ,k-1}$$ (39)

Step 3: Measurement update of *D*_*k*_:

Cubature Points,

$$S_{\theta ,k}=\sqrt{P_{\theta ,k}}$$ (40)

$${\hat{\chi }}_{\theta ,k}^{i}=\theta_{k}+S_{\theta ,k}\xi_{\theta ,i}$$ (41)

$$z_{k}^{i}=g({\hat{\chi }}_{\theta ,k}^{i})+\gamma_{\theta ,k},i=1,2,\ldots ,m$$ (42)

Observation prediction:

$${\hat{z}}_{k}=\frac{1}{m}\sum_{i=1}^{m}z_{k}^{i}$$ (43)

The Kalman gain is as follows:

$$P_{\theta ,y,k}=\frac{1}{m}\sum_{i=1}^{m}(z_{k}^{i}-{\hat{z}}_{k}){\left(z_{k}^{i}-{\hat{z}}_{k}\right)}^{T}+R_{\theta ,k}$$ (44)

$$P_{\theta ,xy,k}=\frac{1}{m}\sum_{i=1}^{m}({\hat{\chi }}_{\theta ,k}^{i}-\theta_{k}){\left(z_{k}^{i}-{\hat{z}}_{k}\right)}^{T}$$ (45)

$$K_{\theta ,k}=P_{\theta ,xy,k}P_{\theta ,y,k}^{-1}$$ (46)

The optimal estimation of state variables is as follows:

$${\hat{\theta }}_{k}=\theta_{k}+K_{\theta ,k}[z_{k}-{\hat{z}}_{k}]$$ (47)

The optimal estimate of the covariance is as follows:

$${\hat{P}}_{\theta ,k}=P_{\theta ,k}-K_{\theta ,k}P_{\theta ,y,k}K_{\theta ,k}^{T}$$ (48)

State error and observation error:

$$\varepsilon_{\theta ,k}={\hat{\theta }}_{k}-\theta_{k}$$ (49)

$$\varepsilon_{z,k}={\hat{z}}_{k}-z_{k}$$ (50)

Step 4: Process noise covariance equation is as follows:

$$q_{\theta ,k}=(1-d_{\theta ,k})q_{\theta ,k-1}+d_{\theta ,k}\varepsilon_{\theta ,k}$$ (51)

$$Q_{\theta ,k}=(1-d_{\theta ,k})Q_{\theta ,k-1}+d_{\theta ,k}[K_{\theta ,k}\varepsilon_{z,k}\varepsilon_{z,k}^{T}K_{\theta ,k}^{T}+P_{\theta ,k}-A_{k-1}{\hat{P}}_{\theta ,k-1}A_{k-1}^{T}]$$ (52)

Step 5: Observation noise covariance equation is as follows:

$$\gamma_{\theta ,k}=(1-d_{\theta ,k})\gamma_{\theta ,k-1}+d_{\theta ,k}\varepsilon_{z,k}$$ (53)

$$R_{\theta ,k}=(1-d_{\theta ,k})R_{\theta ,k-1}+d_{\theta ,k}\varepsilon_{z,k}\varepsilon_{z,k}^{T}-{C_{k}P_{z,k}C}_{k}^{T}]$$ (54)

where $d_{k}=\frac{1-b_{\theta }}{1-b_{\theta ,k}}$, *k* = 1,2,⋯,*n*, *b*_*θ*_ is the forgetting factor,0<*b*_*θ*_<1; *θ*_*k*_, *z*_*k*_, and *P*_*θ*,*k*_ are the estimation of the state, estimation of observation value variable, and estimation of the error covariance; ${\hat{\theta }}_{k},\;{\hat{z}}_{k}$, and ${\hat{P}}_{\theta ,k}$ are optimal estimation of the state variable, actual observation value variable, and optimal estimation of the error covariance.

### 3.3. Optimize OIR and AE based on LSTM

**Fig 3:**
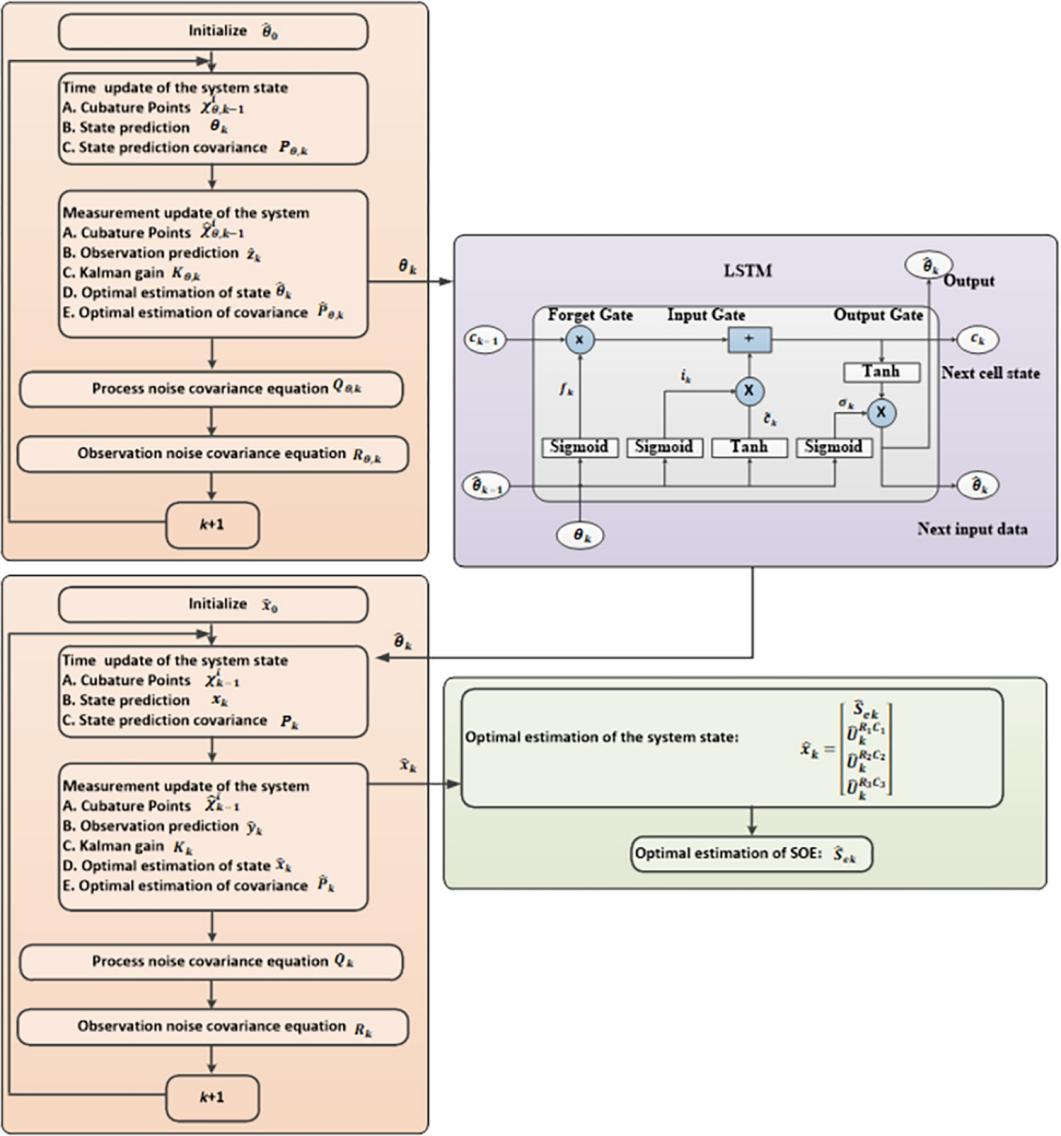
SOE estimation flow chart.

In order to further improve the SOE estimation accuracy, this paper adopts LSTM to optimize OIR and AE in the Kalman filter process [28]. The memory cell structure of LSTM is demonstrated in Fig 3.

$$f_{k}=\sigma (b_{f}+\omega_{f,\hat{\theta }}{\hat{\theta }}_{k-1}+\omega_{f,\theta }\theta_{k})$$ (55)

$$i_{k}=\sigma (b_{i}+\omega_{i,\hat{\theta }}{\hat{\theta }}_{k-1}+\omega_{i,\theta }\theta_{k})$$ (56)

$${\tilde{c}}_{k}=\tanh\left(\omega_{c,\theta }\theta_{k}+\omega_{c,\hat{\theta }}{\hat{\theta }}_{k-1}+b_{c}\right)$$ (57)

$$c_{k}=c_{k-1}*f_{k}+i_{k}*{\tilde{c}}_{k}$$ (58)

$$o_{k}=\sigma (b_{o}+\omega_{o,\hat{\theta }}{\hat{\theta }}_{k-1}+\omega_{o,\theta }\theta_{k})$$ (59)

$${\hat{\theta }}_{k}=o_{k}*tanh(c_{k})$$ (60)

where *θ*_*k*_ and *c*_*k*_ are the input data and status of the memory cell at t time step, ${\hat{\theta }}_{k}$ denotes the output data at the previous time step. *f*_*k*_, *i*_*k*_, and *o*_*k*_ represent forget gate, input gate, and output gate, respectively. This process defined above is repeated at each time step. Additionally, *σ* and * are the sigmoid function and element-wise product. *ω* and *b* are the weight matrices and bias vectors.

In this paper, the main parameters of the LSTM model are as follows: 3 input layer variables; 3 output layer variables; 150 hidden layer units; 1 hidden layer; 200 epochs; and an adjustable parameter, batch_size, of 128.

### 3.4. SOE estimation based on ACKF and LSTM

SOE estimation flow chart of LIB based on ACKF is shown in Fig 3.

## 4. Simulation and discussion

### 4.1. Experiment

**Fig 4:**
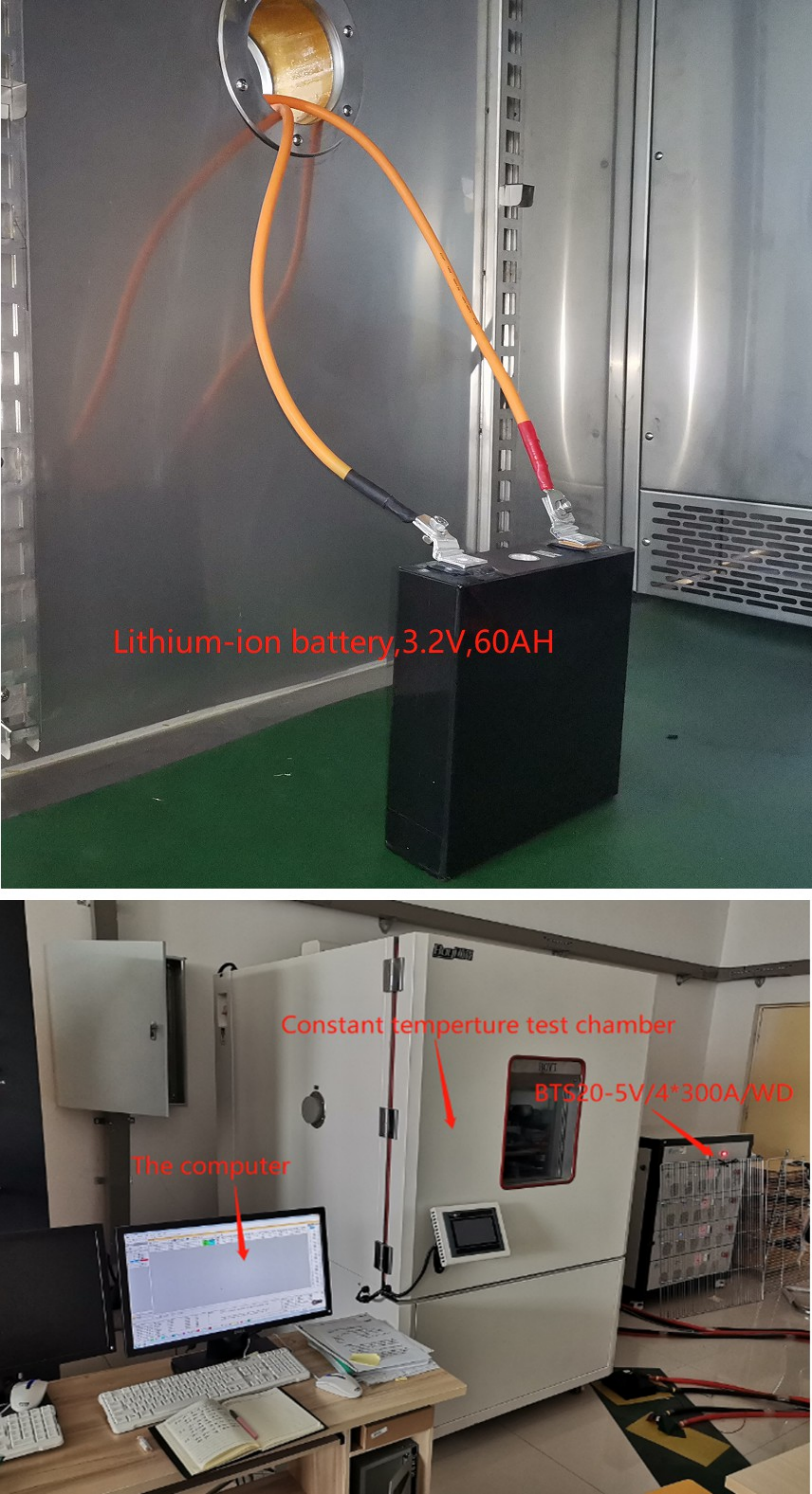
Battery testing system. (**a**) The lithium-ion battery. (**b**) The charge/discharge experiment (BTS20).

The experimental equipment is shown in Fig 4. BTS20 is used to charge and discharge the LIB (as shown in Table 2). First of all, the LIB is charged, and after full, the LIB is discharged several times, and different discharge currents are used. Finally, simulation verification and analysis are carried out based on MATLAB R2023a.

**Table 2:** Parameters of the experiment.

Items	Parameter	Remarks
Capacity of LIB	60 Ah	Ampere hour
Nominal voltage of LIB	3.2 V	Volt
Rated power of LIB	192 W	Wallter
Working voltage of LIB	2.5 V to 3.65 V	
Charging time	3 h	hour
Charging current	20 A	Ampere
Data recording time	1 S	Second
Discharging current	30 A	Ampere
Charging/discharging temperature	25°C	

To verify the adaptive characteristics of LSTM optimization ACKF algorithm, a test experiment was carried out on a fully charged LIB, starting from SOE of 100% to ending at SOE of 25%. In this article, the initial SOE values were changed to 100%, 65%, and 30% separately, and the adaptive and error curves were observed and analyzed.

In the process of simulation verification, the estimated values of SOE were calculated based on LSTM optimization ACKF algorithm, and the actual values were acquired by BTS20.

According to Eq (30), the formula for error is as follows.

The SOE error of LSTM optimization ACKF formula:

$$SOE\;error\;of\;ACKF={\hat{S}}_{ek}-S_{actual}$$ (61)

where, *S*_*actual*_ is the value acquired by the test equipment.

### 4.2. SOE starts at 100%

**Fig 5:**
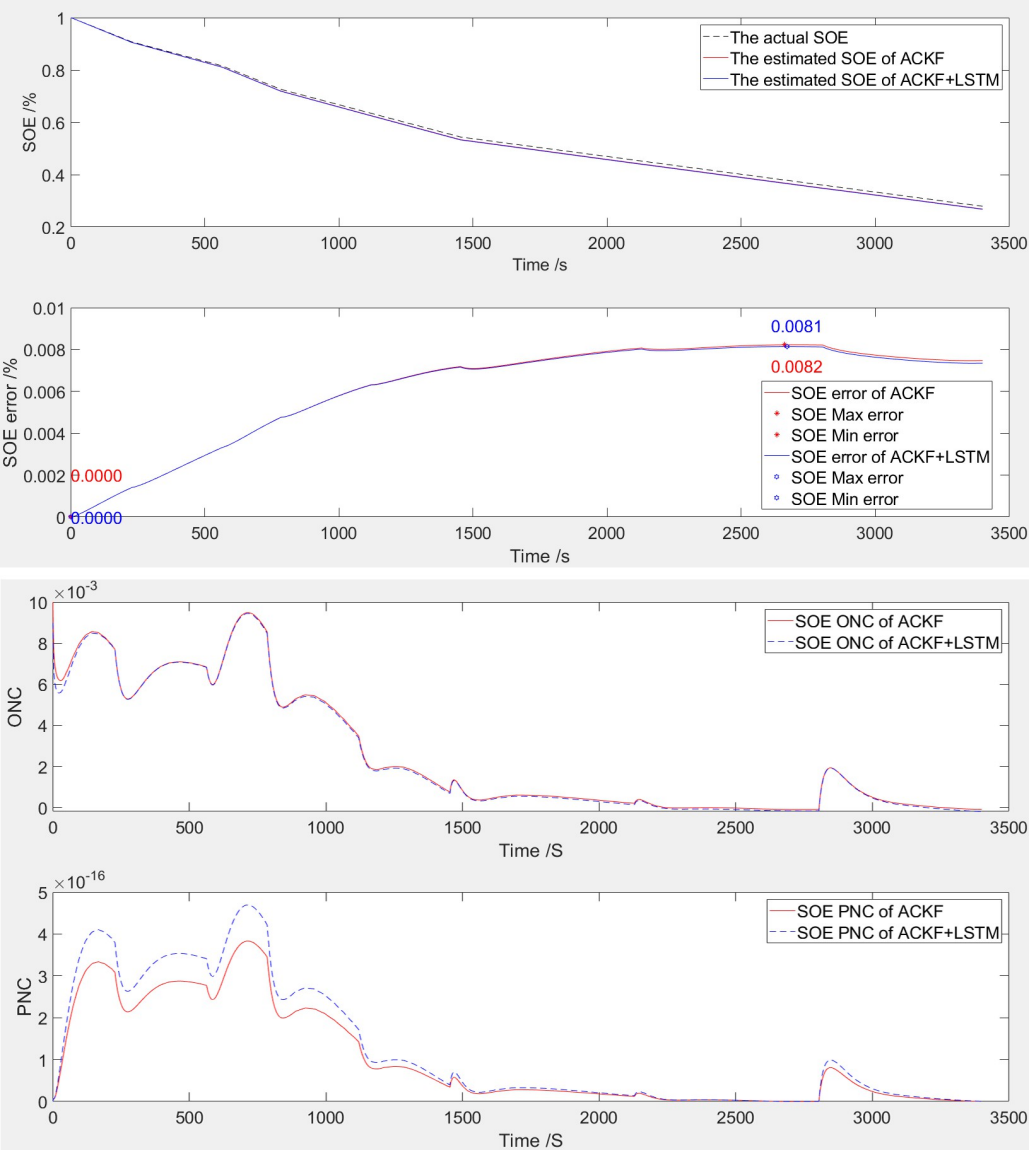
The simulation comparative validation curve when SOE starts at 100%. (**a**) Estimation and error curves of SOE, (**b**) ONC and PNC.

The simulation comparison validation curve of SOE at 100% startup is shown in Fig 5.

The SOE error curve and SOE adaptive estimation curve of LIB are shown in Fig 5A, which were generated through ACKF and LSTM optimized ACKF (ACKF+LSTM). According to Table 5 and Fig 5, the SOE error range based on ACKF ranges from 0% to 0.82%; The SOE error range of ACKF+LSTM is from 0% to 0.83%.

The observation noise covariance (ONC) and process noise covariance (PNC) curves of LIB are shown in Fig 5B. The following figure shows the curve of PNC, and the above figure shows the curve of ONC. Based on the observation and processing of the variation trend of the noise covariance curve, this algorithm has convergence.

### 4.3. SOE starts at 65%

**Fig 6:**
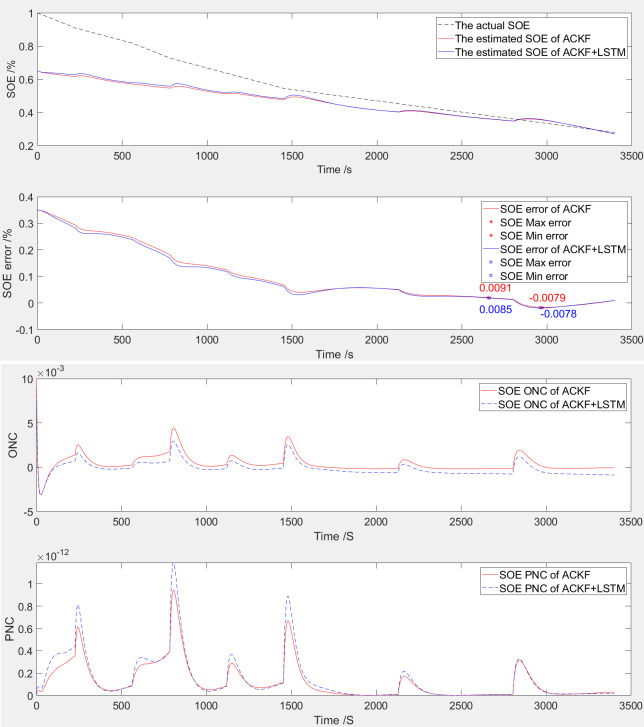
The simulation comparative validation curve when SOE starts at 65%. (a) Estimation and error curves of SOE, (b) ONC and PNC.

The simulation comparison validation curve of SOE at 65% startup is shown in Fig 6.

The SOE estimation curve of LIB is shown in Fig 6A. The following figure shows the SOE error curve, and the above figure shows the SOE adaptive estimation curve. As shown in Table 3 and Fig 6, based on ACKF, the SOE error is -0.79% to 0.91%. Based on ACKF+LSTM, the SOE error is -0.78% to 0.85%.

**Table 3:** Estimation error of LIB’s parameters.

Estimation Error		SOE error of ACKF	SOE error of ACKF+LSTM
SOE = 100%	MAX Error	0.82%	0.81%
	MIN Error	0%	0%
SOE = 65%	MAX Error	0.91%	0.85%
	MIN Error	-0.79%	-0.78%
SOE = 30%	MAX Error	0.93%	0.88%
	MIN Error	-0.82%	-0.82%

Through comparative analysis, the algorithm has good adaptive characteristics.

The ONC and PNC curves of LIB are shown in Fig 6B. The following figure shows the curve of PNC, and the above figure shows the curve of ONC. Based on the observation and processing of the variation trend of the noise covariance curve, this algorithm has convergence. Compared to when the SOE is 100%, the covariance change of observation noise is relatively small, but the covariance change of process noise is relatively large.

### 4.4. SOE starts at 30%

**Fig 7:**
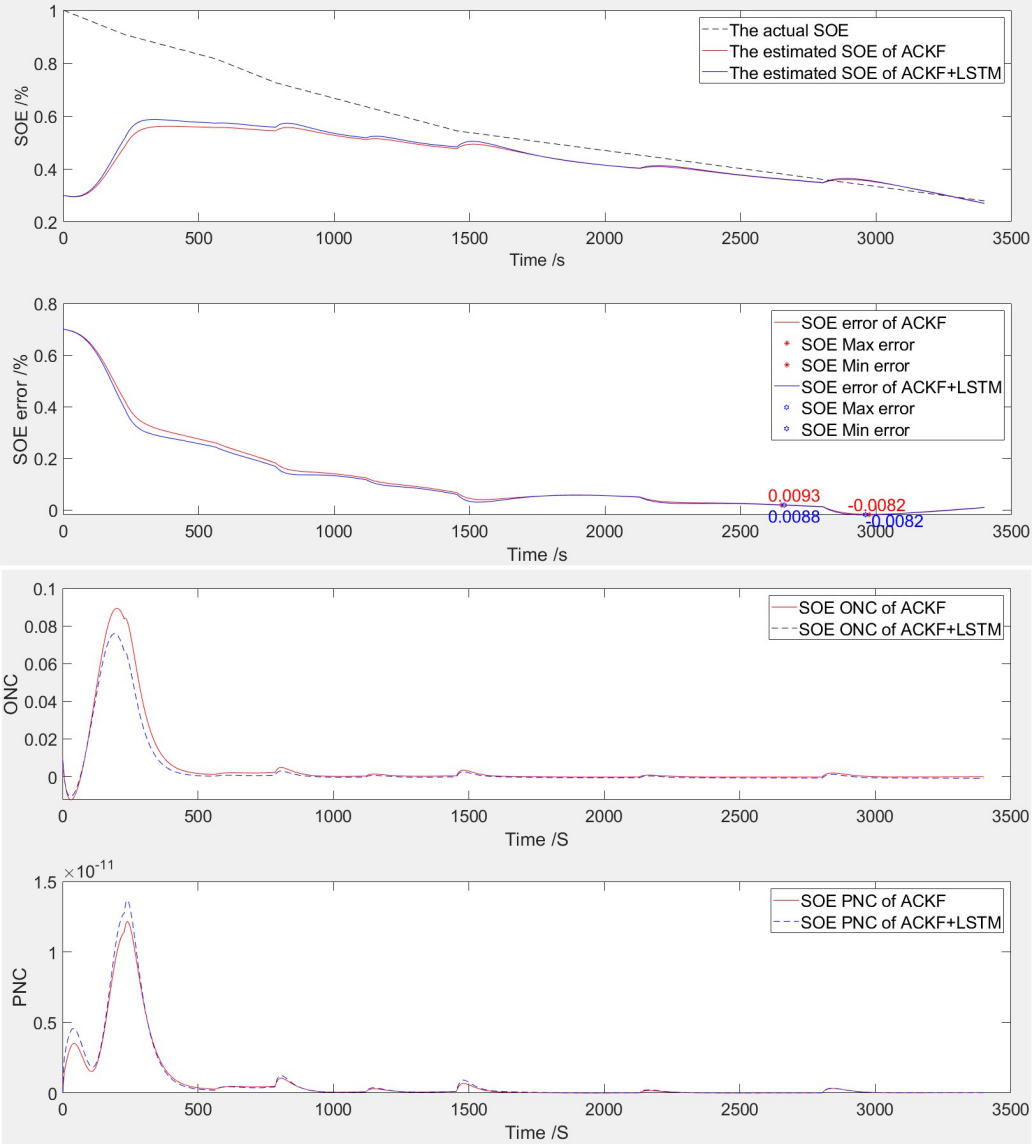
The simulation comparative validation curve when SOE starts at 30%. (a) Estimation and error curves of SOE, (b) ONC and PNC.

The simulation comparison validation curve of SOE at 30% startup is shown in Fig 7.

The SOE estimation curve of LIB is shown in Fig 7A. The following figure shows the SOE error curve, and the above figure shows the SOE adaptive estimation curve. As shown in Table 3 and Fig 7, based on ACKF, the SOE error is -0.82% to 0.93%. Based on ACKF+LSTM, the SOE error is -0.84% to 0.90%. With the increase of the initial error, the difference of standard deviation increases, which accords with the statistical law.

The ONC and PNC curves of LIB are shown in Fig 7B. The following figure shows the curve of PNC, and the above figure shows the curve of ONC. Based on the observation and processing of the variation trend of the noise covariance curve, this algorithm has convergence. Compared to when the SOE is 65%, both the observed values and the covariance of process noise have increased.

### 4.5. Discussion

The simulation results show that the method based on ACKF+LSTM has an SOE estimation error of less than 0.88% for LIB regardless of SOE at 100%, 65%, and 30%, which is more accurate than the SOE estimation error of the individual ACKF.

As shown in Table 4, compared with accuracy of 1.19% in Ref. [29], 2% in Ref. [30], 1.93% in Ref. [31], 2.34% in Ref. [6], the method has high precision. In addition, the combination method is self-adaptive.

**Table 4:** Comparison of methods.

Reference	Accuracy of estimation	Adaptability	Methods	Limitations
Method of this paper	0.88%	Yes	ACKF+LSTM	Large amount of calculation
[29]	1.19%	Yes	LSTM + AEKF	Large amount of calculation
[6]	2.34%	Yes	AUKF	Low accuracy
[30]	2%	Yes	EKF	Low accuracy
[31]	1.93%	Yes	Fuzzy+ACKF	Large amount of calculation, low accuracy

As shown in Table 5, by comparing MSE (Mean square Error), MAE (Mean Absolute Error), RMSE (Root Mean square Error) and SD (Standard Deviation), it can be seen that, the method of ACKF+LSTM is more advantageous.

**Table 5:** Statistical error of methods.

Error		error of ACKF	error of ACKF+LSTM
SOE = 100%	MSE	4.4094×10^−5^	4.4064×10^−5^
	MAE	2.5358×10^−6^	2.5226×10^−6^
	RMSE	0.0066404	0.0066304
	SD	0.2036	0.2033
SOE = 65%	MSE	0.0045549	0.0045449
	MAE	3.4453×10^−5^	3.1569×10^−5^
	RMSE	0.06749	0.06629
	SD	0.1034	0.0999
SOE = 30%	MSE	0.0096851	0.0096751
	MAE	4.0816×10^−5^	3.6813×10^−5^
	RMSE	0.098413	0.098113
	SD	0.0926	0.0864

The estimation method based on ACKF+LSTM is superior to using ACKF alone in terms of prediction accuracy and stability. By using ACKF+LSTM, the accuracy of SOE estimation has been significantly improved, which is of great significance for real-time battery management systems.

## 5. Conclusions

This article establishes a SOE estimation method based on ACKF+LSTM. In order to improve the accuracy of SOE estimation, LSTM is introduced on the basis of adaptive Kalman filter to optimize OIR and AE, and ACKF and LSTM are combined. Through this method, we can better handle and predict complex nonlinear dynamic systems, and improve the accuracy of SOE estimation. In addition, the proposed SOE estimation method was experimentally validated, and the experimental results showed that the SOE estimation method based on ACKF+LSTM has higher accuracy and robustness.

This paper has made some achievements, there are still many deficiencies: (1) In this paper, the number of samples is not enough, the next step will increase the number of samples, test universality. (2) parameter identification is offline at the moment; the next step will be to carry out the parameter identification of online identification.

## Supporting information

S1 Data (ZIP)

## Abbreviations

The following abbreviations are most common in this manuscript: SOE: State of energy
LIB: Lithium-ion battery
ACKF: Adaptive Cubature Kalman Filter
OIR: Ohmic internal resistance
AE: Actual energy
LSTM: Long short-term memory
TRCEM: Third-order Resistor-Capacitance equivalent model
BMS: Battery management system
SVD: Singular value decomposition
FFRLS: Forgetting factor recursive least squares
OBE: Optimal bounding ellipsoid
LKF: Linear Kalman filter
VFFRLS: Variable forgetting factor recursive least squares
WMICKF: Weighted multi-innovation cubature Kalman filter
OCV: open circuit voltage
RNN: Recurrent neural network
BTS20 5V/4*300A/WD: Hubei Techpow Electric Co., Ltd., China
